# The idiopathic intracranial hypertension prospective cohort study: evaluation of prognostic factors and outcomes

**DOI:** 10.1007/s00415-022-11402-6

**Published:** 2022-10-15

**Authors:** Mark Thaller, Victoria Homer, Yousef Hyder, Andreas Yiangou, Anthony Liczkowski, Anthony W. Fong, Jasvir Virdee, Rachel Piccus, Marianne Roque, Susan P. Mollan, Alexandra J. Sinclair

**Affiliations:** 1 Translational Brain Science, Institute of Metabolism and Systems Research, University of Birmingham, Birmingham, B15 2TT UK; 2 Department of Neurology, University Hospitals Birmingham NHS Foundation Trust, Birmingham, B15 2TH UK; 3 Centre for Endocrinology, Diabetes and Metabolism, Birmingham Health Partners, Birmingham, B15 2TH UK; 4 Cancer Research (UK) Clinical Trials Unit, University of Birmingham, Birmingham, B15 2TT UK; 5 Emergency Medicine, Hull University Teaching Hospitals NHS Trust, Anlaby Rd, Hull, HU3 2JZ UK; 6 Birmingham Neuro-Ophthalmology, University Hospitals Birmingham NHS Foundation Trust, Birmingham, B15 2TH UK; 7 Ophthalmology, School of Medicine, University of Queensland, Queensland, 4006 Australia

**Keywords:** Pseudotumor cerebri, Vision, Headache, Optical coherence tomography, Outcome, Prognosis, Idiopathic Intracranial Hypertension

## Abstract

**Background:**

There are limited longitudinal data evaluating outcomes in idiopathic intracranial hypertension (IIH). We aimed to evaluate the long-term outcomes in a real-world cohort of patients with IIH and sought to establish the prognostic factors.

**Methods:**

A longitudinal prospective cohort study was conducted over 9 years (2012–2021). Data included demographics and disease status. All consenting patients with IIH were recruited. Visual outcomes included visual acuity, Humphrey visual field and optical coherence tomography (OCT) imaging measurements. Headache frequency, severity, and impact were noted. We analysed the key variables impacting visual and headache outcomes.

**Results:**

The cohort contained 490 patients with a confirmed IIH diagnosis. 98% were female with a mean body mass index (BMI) of 38 kg/m^2^. Those with the highest OCT retinal nerve fibre layer had the worst visual outcomes. We noted a delayed decline, in the visual field and OCT ganglion cell layer after 12 months. In the medically managed cohort (*n* = 426), we found that disease duration and change in BMI had the greatest influence on visual outcomes. There was a high burden of headache, with a daily headache at presentation and prior migraine history influencing long-term headache prognosis.

**Conclusions:**

There is a delayed decline in visual outcomes in those with the most severe papilloedema. Disease duration and change in BMI were the key visual prognostic factors, therefore those with the more acute disease may require closer monitoring. Improving prognosis in IIH should focus on the potentially modifiable factor of weight management.

## Introduction

Idiopathic intracranial hypertension (IIH) predominately affects women with obesity and emerging evidence suggests it is a neuro-metabolic disorder [1–3]. It is a condition of raised intracranial pressure (ICP) which can lead to chronic headaches, visual loss and cognitive dysfunction [2, 4, 5]. It has a number of comorbid conditions such as obstructive sleep apnoea, infertility, increased pregnancy complications, and long-term risk of adverse cardiovascular outcomes [6–8]. For the majority of patients the visual prognosis is good. However, poor visual prognostic factors that have previously been demonstrated include male sex, ethnicity, high-grade papilloedema, > 30 transient visual obscurations per month and decreased visual acuity at baseline [9–11]. Headaches associated with IIH are problematic, debilitating, and persistent [12–14]. Weight loss is currently the only known disease modifier in IIH [15] and the recent IIH Weight Trial showed that bariatric surgery was superior over community weight management in lowering the intracranial pressure [16] with it also being cost-effective [17]. Maintenance of weight loss especially by dietary means can be difficult [3]. Reports state that IIH relapse is common, occurring between 9% and 28% in IIH patients [13, 18, 19]. Pharmacological interventions currently consist of off-label use of acetazolamide, topiramate and other diuretics [5, 20]. When papilloedema is sight threatening, surgical intervention is often required [5, 21, 22].

Within the literature, there are few large cohorts describing the longitudinal course of IIH [13, 23–25]. Most were performed retrospectively or focus on particular interventions[26, 27] or investigate specific risks such as sex or ethnicity [11, 28]. There is a need for large prospective cohort studies evaluating the real-world clinical course of IIH. Whilst there has been a number of randomised controlled trials and small case–controlled cohort studies, these define specific populations for their inclusion criteria such as new onset disease (as in the IIH Treatment Trial) [20, 29, 30] or chronic disease (as in the IIH Weight Trial) [16] and therefore do not focus on the whole disease spectrum. We aimed to evaluate the long-term outcomes in a prospectively collected real-world cohort of patients with IIH irrespective of disease timing. We hypothesised that those with the greatest papilloedema would have worse visual outcomes, and those with the greatest burden of headache at the start of the disease may have a more challenging clinical course. We evaluated all patients but also aimed to determine the key prognostic indicators in those managed exclusively medically (no IIH surgical interventions).

## Methods

### Study poplation

The IIH:Life study is an ongoing prospective observational cohort study of patients with IIH attending a specialist clinic at a single tertiary neuroscience centre in the United Kingdom [University Hospitals Birmingham NHS Foundation Trust (UHB)]. Data presented in this manuscript was prospectively collected at the time of their routine clinical visits over a 9-year period (between April 23rd 2012 and Sep 8th 2021). All sequential consenting patients were included, and written informed consent obtained from all the patients.

### Diagnosis of IIH

Patients eligible for inclusion were those with a confirmed diagnosis of IIH as per the modified Dandy criteria: this includes papilloedema, neuroimaging excluding a venous sinus thrombosis or structural lesion, lumbar puncture opening pressure > 25 cmCSF in a properly performed procedure [31]. Patients were enrolled at their first visit to the neuro-ophthalmology outpatient clinic. They were either initially diagnosed at UHB or at another referring hospitals within the United Kingdom. Those who were referred with a potential diagnosis of IIH but in whom the diagnosis was not confirmed, those with a secondary cause of intracranial hypertension, and those with IIH without papilledema (IIHWOP) were excluded from the study. The medically treated cohort was evaluated as were those managed without surgical intervention (defined as a cerebrospinal fluid (CSF) diversion, optic nerve sheath fenestration (ONSF) or venous sinus stenting intervention for IIH conducted at any time).

### Data collection

The following data was collected: dates of clinical visits, date of first diagnostic lumbar puncture (used to as a surrogate marker of disease duration, defined as the time from the first diagnostic lumbar puncture to baseline clinical visit at the IIH clinic), Body mass index (BMI), diagnostic lumbar puncture opening pressure, ICP medication prescription [acetazolamide, topiramate, other diuretics (furosemide and amiloride)] and details regarding any surgeries for IIH. Visual outcomes included visual acuity [measured using Logarithm of the Minimum Angle of Resolution (LogMAR)] and perimetric mean deviation [PMD; Humphrey 24–2 (Swedish Interactive Testing Algorithm (SITA) central threshold)]. Papilloedema measures included Frisén grading (0–5 where 0 equates to no papilledema and 5 where all vessels are at least partially obscured by oedema) [32, 33] as established by a suitably trained neuro-ophthalmology specialist following dilated slit lamp examination; and optical coherence tomography (OCT) imaging (Heidelberg Spectralis™) parameters of average global peripapillary retinal nerve fibre layer (RNFL); a manually measured total retinal thickness (TRT); and macular ganglion cell layer (GCL) volume (1, 2.22, 3.45 mm volume scan), by macular volume and/or posterior pole methods. Automated segmentation of retinal layers by OCT software may have inaccuracies in moderate to severe papilloedema [34]. To ensure accuracy, manual segmentation of RNFL and TRT was performed in peripapillary scans, in addition to manual segmentation of the basement membrane (BM) and inner limiting layer (ILM) for cross-sectional slices of optic disc scans where appropriate.

Headache outcomes were evaluated by assessing headache frequency (monthly headache days), migraine-like headache frequency (monthly migraine-like headache days) [35], headache severity (0–10 numerical rating scale, where 0 is no pain and 10 equates to the most severe pain), and headache disability using the Headache Impact Test-6 (HIT-6) test [36]. Details of the presence of daily headache at baseline (defined as headache days of ≥ 28 days/month) [35], prior history of migraine, familial history of migraine, and analgesic use were also collected as these were felt to be factors that could potentially influence headache prognosis.

### Disease status definition

At the baseline clinical visit, following confirmation of a diagnosis of IIH, disease status was determined. Active IIH was defined as those with active papilloedema Frisén grade ≥ 1 in at least one eye and IIH in ocular remission was defined as those with no evidence of papilloedema.

IIH relapse was defined as a change from Frisen 0 to 1 based on RNFL measures from ≤ 100 µm to ≥ 109 µm in the same eye [33].

### Statistical analysis

Continuous and categorical variables were reported as mean [standard deviation (SD)] and number (percentage), respectively. Statistical analyses were performed using R (v4.1.0) [37].

To assess longitudinal visual outcomes, the effects of disease status at the time of entry to the IIH:Life study (either active IIH or IIH ocular remission); effect of time from the first lumbar puncture to enrolment (as the proxy for disease duration); lumbar puncture opening pressure at registration (both continuously and categorised < 25, 25–29.9,30–39.9, or > 40 cmCSF); BMI at first visit; BMI at each visit; and age at the first visit. Models were developed independently for each visual outcome using forward stepwise regression, with our null models adjusting for disease status and ICP medication, as our a priori hypothesis was that such factors were likely to have the greatest effect on visual outcomes.

Regression models were fitted using lme4 [38]. We assumed the continuous form of our dependent variables for all outcomes. Population-level terms were used to estimate the average response value, an adjustment for a time from study registration to outcome measure, and an interaction between variables of interest and time. Patient-level intercepts were included to address serial correlation in responses, and the nesting of measurements from eyes within a patient (as modelling included data from both eyes, where available). Where covariates were added to models, they were transformed or centred around the median value as appropriate. Models were developed independently for each visual outcome.

For headache outcomes, in those with active IIH initially, the effect of any surgical intervention was explored. Further analyses then explored the effects of medication overuse, previous personal migraine history, familial history of migraine, presence of daily headache at registration, disease duration, and BMI. Analogous processes and models to those employed for visual outcomes were used for headache outcomes, with independent models developed for each headache outcome.

Locally weighted scatterplot smoothing (LOESS) graphs were constructed prior to regression analysis to ascertain the relationship between variables and any emergent trends.

### Patient and public involvement

IIHUK, a national patient charity (Registered Charity in England and Wales no 1143522 & Scotland SCO43294) that supports carers and patients with IIH endorsed and helped develop the IIH Life concept and questionnaire. IIHUK contributed to funding the project. The Medical Research Council (MRC) MR/KO15184/1, National Institute of Health Research (NIHR) Healthcare Quality Improvement Partnership (HQIP) grant NIHR-CS-011-028 and the Sir Jules Thorne Award for biomedical science have helped fund this project.

## Results

**Fig. 1:**
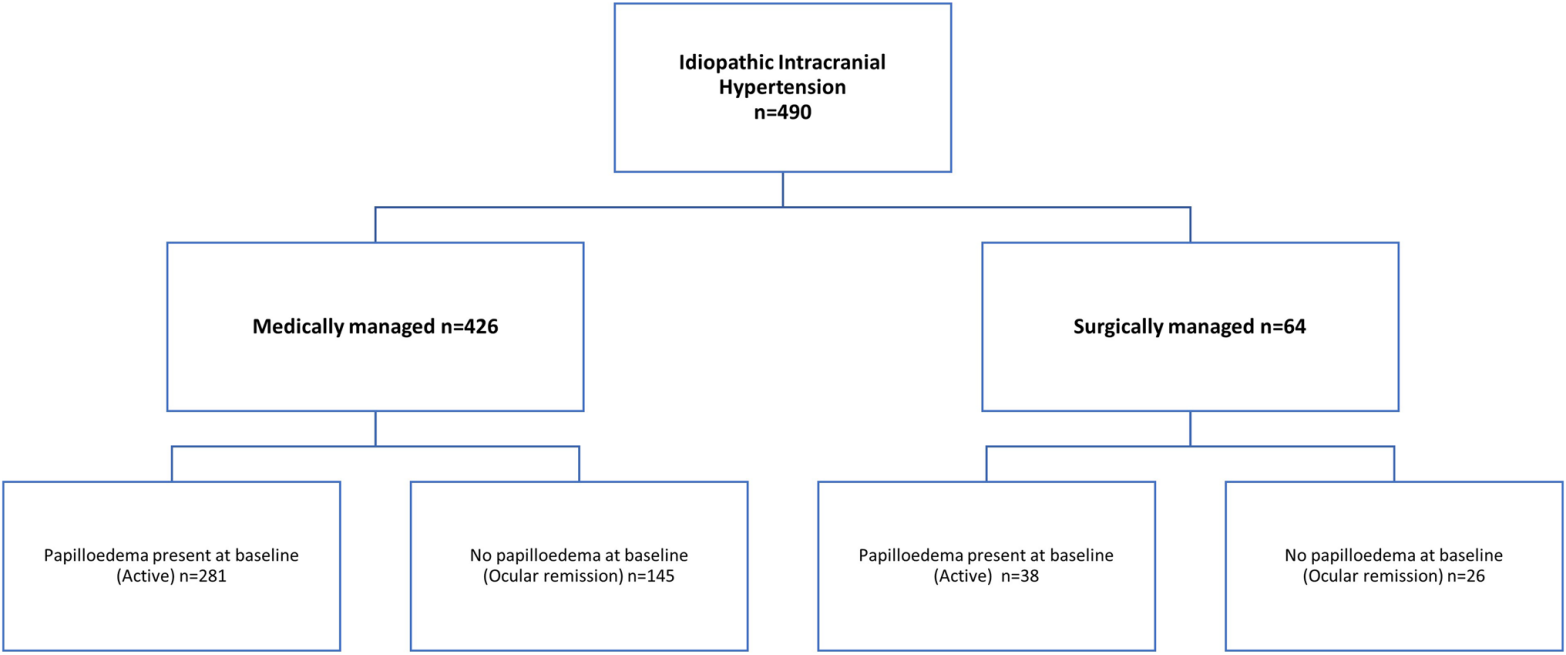
CONSORT diagram

This analysis includes 490 patients with a confirmed diagnosis of IIH, with a mean disease duration of 17 (range 0–312) months. 161 patients had a single visit, with 329 having multiple visits (mean 4 visits; range 2–17 encounters). Those who attended more than once had a mean duration of follow-up of 23.5 (range 0–87) months. Baseline characteristics noted that there were very few men within this cohort (*n* = 9), with the ratio of women: men being 53:1 (Table 1). In the medically managed cohort (*n* = 426), at their first visit 281 had active IIH and 145 were noted to have IIH in ocular remission (Fig. 1). In the surgery cohort, 38 at the first visit all had active IIH and 26 were in ocular remission (but either had surgery prior to enrolment or subsequent due to relapse) (Fig. 1).

**Table 1:** Baseline demographics table

	All IIH	Medically managed	Surgically managed
Total number (*n*)	490	426	64
Sex			
Female (*n*)	481	420	61
Male (*n*)	9	6	3
Status			
Active (*n* (%))	319 (65%)	281 (66%)	38 (59%)
Ocular remission (*n* (%))	171 (35%)	145 (34%)	26 (41%)
Age, years (mean (SD))	31.5 (9.4)	31.6 (9.5)	30.6 (9.0)
BMI, kg/m^2^ (mean (SD))	38.1 (9.3)	37.8 (9.5)	40.2 (7.9)
Weight, kg (mean (SD))	103.0 (26.7)	101.9 (26.9)	110.2 (24.5)
CSF opening pressure, cmCSF [mean (SD)]	36.5 (6.0)	34.7 (4.5)	35.3 (7.1)
Disease duration, months [mean (SD)]	17.2 (36.8)	16.0 (32.0)	26.5 (62.0)

Prescriptions of ICP-lowering medications at first visit included acetazolamide (*n* = 131, 27%; mean dose 1032 mg/day, range 250 mg to 4 g daily), topiramate (*n* = 44, 9%; mean dose 135 mg/day, range 25 to 400 mg daily), and other diuretics (*n* = 21, 4%). Prescriptions of ICP-lowering medications during follow-up were noted of acetazolamide (*n* = 115, 23%), topiramate (*n* = 51, 10%), and other diuretics (furosemide *n* = 1, 0.2%). Analgesic use was common at the first visit (*n* = 224, 46%) with some patients using more than one class. Prescriptions of the following analgesic medications were noted at baseline visit: paracetamol (*n* = 129, 24%), ibuprofen (*n* = 64, 13%), triptan (*n* = 57, 11%), co-codamol (*n* = 48, 9%), naproxen (*n* = 31, 6%) and tramadol (22, 4%). In the sub-group of patients that reported headache on more than 15 days of the month (*n* = 111, 23%), the analgesic use was higher: paracetamol (*n* = 40, 36%), triptan (*n* = 35, 32%), ibuprofen (*n* = 19, 17%), co-codamol (*n* = 20, 15%) and naproxen (*n* = 11, 10%). Medication overuse was recorded in 18% of patients at baseline (*n* = 87), with commonest culprit medications being paracetamol (*n* = 49, 56%), opiates (*n* = 36, 41%) and ibuprofen (*n* = 22, 25%). Migraine prophylaxis at baseline was recorded with topiramate (*n* = 73, 14%), amitriptyline (*n* = 59, 11%) and propranolol (*n* = 28, 5%) having been prescribed.

IIH relapse was only noted in 18 (3.7%) patients whilst under follow-up and only 2 of these had previously been in ocular remission at baseline visit.

### Factors affecting long-term visual outcomes in IIH

**Fig. 2:**
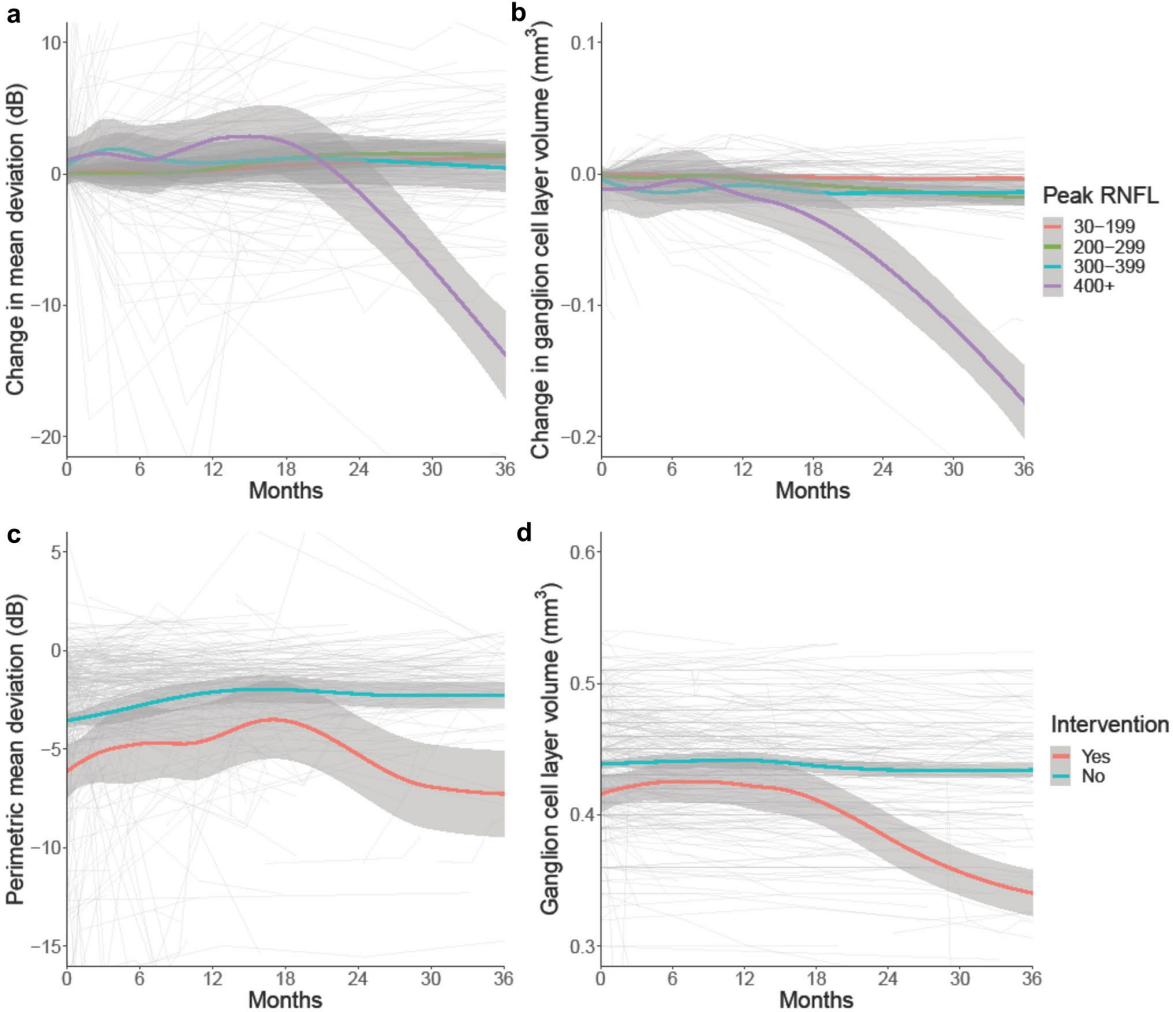
**a** Longitudinal data of Humphrey visual field perimetric mean deviation (PMD) in IIH categorised by peak global retinal nerve fibre layer thickness, and LOESS smoothers added to show trends across the categories. **b** Longitudinal data of macular ganglion cell layer (GCL) volume in IIH categorised by peak global retinal nerve fibre layer thickness, and LOESS smoothers added to show trends across the categories. **c** Longitudinal data of Humphrey visual field perimetric mean deviation (PMD) in IIH categorised by surgical intervention or not, and LOESS smoothers added to show trends across the categories. **d** Longitudinal data of macular ganglion cell layer (GCL) volume in IIH categorised by surgical intervention or not, and LOESS smoothers added to show trends across the categories

The most prominent factor influencing long-term visual function (as measured by PMD) was the extent of papilloedema measured by the highest global RNFL on OCT for individual patients during the course of follow-up. Of note the decline in PMD mirrored the reduction in the macular GCL volume (Fig. 2), in particular for those with RNFL ≥ 400 µm or TRT ≥ 800 µm. However, the GCL and PMD decline was delayed and was not observed until greater than 12 months following the first encounter.

The cohort was then dichotomised into those medically or surgically managed. Amongst those who were medically managed the GCL volume remained stable over time. Whilst in those who underwent a surgical intervention there was a worse prognosis where both the PMD and macular GCL volume significantly declined after 12 months (Table 2; Fig. 2).

**Table 2:** Impact of surgical intervention over time (months) on perimetric mean deviation (PMD) and macular ganglion cell layer volume (GCL)

	0	6	12	24	36
Humphrey visual field perimetric mean deviation, dB (mean (SD), n)					
Intervention	− 6.20 (4.59), 45	− 4.45 (4.24), 19	− 4.51 (3.65), 16	− 4.89 (3.24), 10	− 9.70 (4.18), 8
No intervention	− 3.55 (3.41), 351	− 2.77 (4.40), 168	− 2.02 (3.95), 134	− 2.19 (3.05), 90	− 2.18 (3.18), 49
Macular ganglion cell layer volume, mm^3^ (mean (SD), n)					
Intervention	0.414 (0.035), 24	0.429 (0.031), 11	0.421 (0.027), 9	0.384 (0.022), 6	0.321 (0.028), 6
No intervention	0.439 (0.026), 224	0.441 (0.031), 116	0.443 (0.027), 92	0.434 (0.022), 64	0.436 (0.023), 42

### Factors affecting long-term visual outcomes in medically managed IIH

**Fig. 3:**
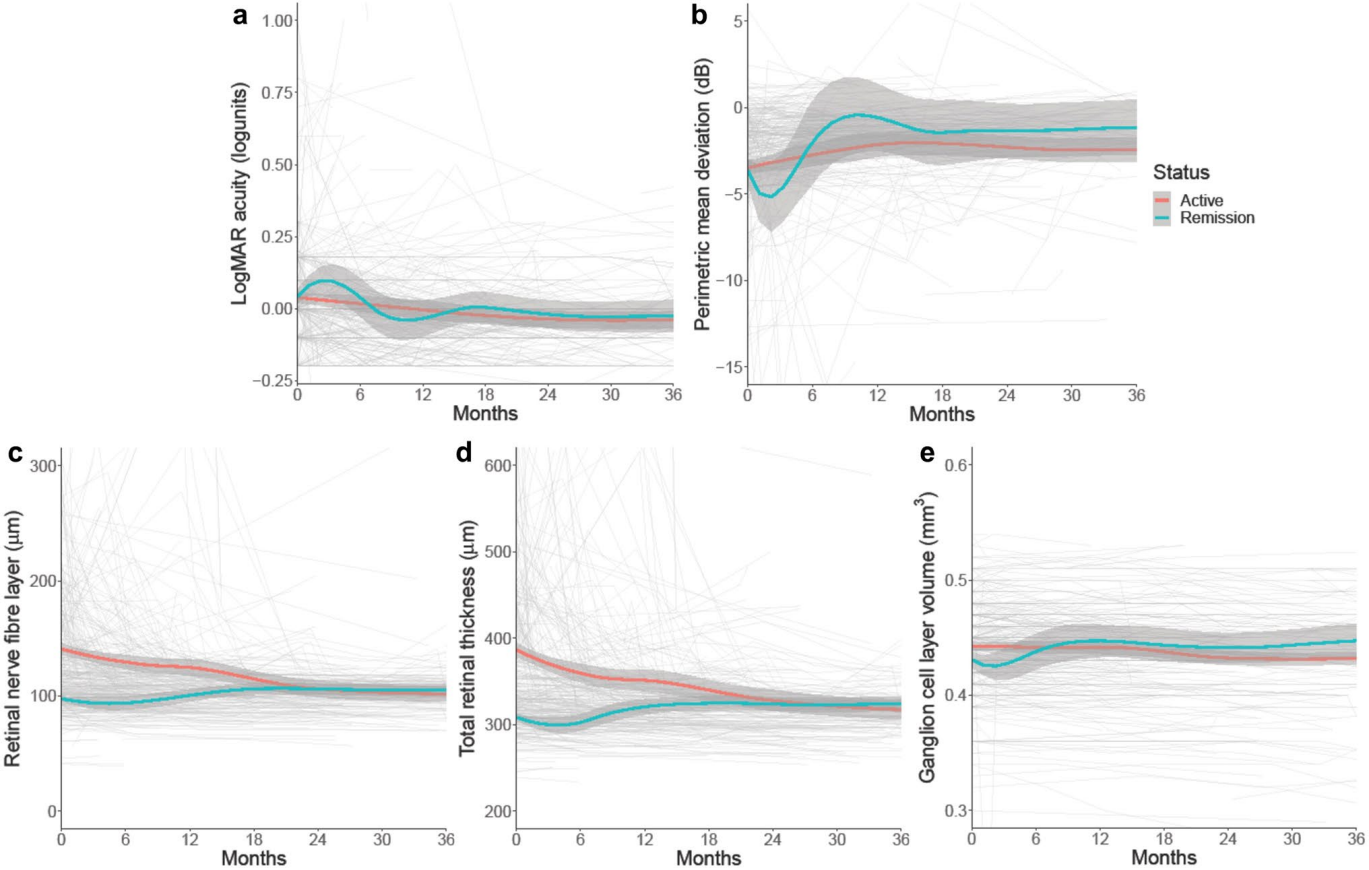
Longitudinal visual data from medically managed IIH patients categorised by disease status—active disease (papilloedema present at enrolment) versus ocular remission (no papilloedema at enrolment), and LOESS smoothers added to show trends across the categories. **a** LogMAR visual acuity (log units). **b** Perimetric mean deviation measured by Humphrey visual field 24–2 testing (dB). **c** Retinal nerve fibre layer thickness measured on Optical Coherence Tomography (µm). **d** Total retinal thickness of optic nerve head measured on Optical Coherence Tomography (µm). **e** Macular ganglion cell layer volume measured on Optical Coherence Tomography (mm^3^)

Favourable visual outcomes were noted in the sub-group of IIH patients that were medically managed (*n* = 426; Table 3). Visual acuity was minimally affected in this large sub-group and remained similar over time (Table 3). The presence or absence of papilloedema (for those with active IIH and IIH in ocular remission, respectively) at the first encounter had no apparent influence on long-term visual acuity (Table 4; Fig. 3). PMD for the medically managed cohort was initially observed to improve over the first 12 months following enrolment with subsequent stabilisation (Fig. 3B). There was little difference in the trajectory of PMD over time between those initially seen with active IIH versus IIH in ocular remission (Table 4). Patients with active IIH at the first visit had a higher mean RNFL compared to those in remission (active IIH 137.42 µm (95% Confidence Interval (CI) 132.31, 142.54); IIH in ocular remission 97.45 µm (95% CI 89.87, 105.02)) (Table 4; Fig. 3C). The trajectory in the active IIH group reduced by − 1.27 µm/month (95% CI − 1.54, − 1.00) as compared to minimal change in the IIH ocular remission group 0.037 µm/month (95% CI − 0.54, 0.61) (Table 4). At 40 months post-enrolment, RNFL thickness was comparable between these groups. As expected, the TRT showed a similar trend to the RNFL (Table 4; Fig. 3D) with the trajectory in the active IIH group reducing by − 2.63 µm/month (95% CI − 3.09, − 2.17), as compared to minimal change in the IIH ocular remission group of 0.25 µm/month (95% CI − 0.68, 1.17).

**Table 3:** Visual and headache outcomes for medically managed IIH patients

	Months				
	0	6	12	24	36
LogMAR visual acuity, logunits (mean (SD), *n*)	0.04 (0.16), 337	0.02 (0.21), 175	0.00 (0.16), 147	− 0.03 (0.13), 96	− 0.04 (0.14), 59
Humphrey visual field perimetric mean deviation, dB (mean (SD), *n*)	− 3.38 (3.44), 351	− 2.72 (4.50), 168	− 2.03 (4.01), 134	− 2.19 (3.08), 90	− 2.18 (3.22), 49
Global peripapillary retinal nerve fibre layer, µm (mean (SD), *n*)	127.3 (39.0), 277	118.6 (45.8), 136	120.8 (40.0), 110	106.4 (31.3), 74	103.4 (33.1), 48
Global peripapillary total retinal thickness, µm (mean (SD), *n*)	360.5 (56.2), 277	342.5 (64.8), 136	343.1 (57.4), 110	327.2 (44.6), 74	318.1 (48.9), 48
Macular ganglion cell layer volume, mm^3^ (mean (SD), *n*)	0.439 (0.031), 327	0.441 (0.035), 151	0.443 (0.030), 118	0.434 (0.024), 81	0.436 (0.026), 51
Headache frequency, days/month (mean (SD), *n*)	21.0 (12.2), 304	19.2 (14.0), 146	19.1 (12.4), 127	16.6 (10.2), 85	16.1 (11.4), 53
Migraine-like headache frequency, days/month (mean (SD), *n*)	10.0 (8.6), 224	8.8 (9.5), 104	7.9 (9.1), 94	8.4 (7.3), 67	8.0 (8.2), 45
Headache severity, visual analogue scale 0–10 (mean (SD), *n*)	6.3 (2.9), 304	5.7 (3.6), 154	5.8 (3.0), 130	5.9 (2.6), 89	5.5 (2.9), 58
Headache Impact Test 6, score 36–78 (mean (SD), *n*)	55.7 (15.7), 197	57.6 (17.6), 107	55.7 (17.5), 107	58.6 (14.6), 76	59.5 (15.7), 44

**Table 4:** Baseline and trajectory comparison for visual outcomes in active and ocular remission IIH calculated through regression modelling

	Baseline		Trajectory	
	Active	Ocular remission	Active (units/month)	Ocular remission (units/month)
LogMAR visual acuity, logunits	0.025 (95% CI 0.003, 0.047)	0.044 (95% CI 0.013, 0.075)	− 0.0007 (95% CI − 0.0014, − 0.0001)	− 0.0012 (95% CI − 0.0029, 0.0004)
Humphrey visual field perimetric mean deviation, dB	− 3.37 (95% CI − 3.96, − 2.78)	− 3.39 (95% CI − 4.27, − 2.51)	+ 0.027 (95% CI 0.004, 0.050)	+ 0.071 (95% CI 0.021, 0.120)
Global peripapillary retinal nerve fibre layer, µm	137.42 (95% CI 132.31, 142.54)	97.45 (95% CI 89.87, 105.02)	− 1.27 (95% CI − 1.54, − 1.00)	+ 0.037 (95% CI − 0.54, 0.61)
Global peripapillary total retinal thickness, µm	384.86 (95% CI 376.17, 393.54)	308.27 (95% CI 295.85, 320.7)	− 2.63 (95% CI − 3.09, − 2.17)	+ 0.246 (95% CI − 0.68, 1.17)
Macular ganglion cell layer volume, mm^3^	0.440 (95% CI 0.434, 0.446)	0.427 (95% CI 0.419, 0.436)	− 0.0005 (95% CI − 0.0006, − 0.0003)	− 0.0002 (95% CI − 0.0004, 0.0001)

Considering the excellent correlation between the macular volume and posterior pole measures of macular GCL (mm^3^) within our cohort (Pearson correlation 0.98 (95% CI 0.97, 0.98), we combined these analogous measures. At a patients’ first visit GCL volume was significantly reduced for those in ocular remission (0.43 mm^3^ (95% CI 0.42, 0.44)) compared to those with active IIH (0.44 mm^3^ (95% CI 0.43, 0.45), Table 4). We also noted that the duration of the disease was longer in those in ocular remission (mean 32 months (standard deviation (SD) 58)) compared to those who were active (mean 13 months (SD 29)). Long-term analysis revealed GCL volume stability over time with no significant difference between the active and ocular remission in enrolment groups (Fig. 3E).

A stepwise regression evaluated which additional factors affected visual (visual acuity, PMD, RNFL, TRT and GCL volume) prognosis. The change in BMI was the most influential factor affecting PMD, RNFL and macular GCL volume outcomes (Table 5). Whilst diagnostic lumbar puncture opening pressure, disease duration, baseline BMI were less influential (Table 5).

**Table 5:** Factors affecting visual prognosis in medically managed IIH cohort

	LogMAR visual acuity, logunits	Humphrey visual field perimetric mean deviation, dB	Global peripapillary retinal nerve fibre layer, µm	Global peripapillary total retinal thickness, µm	Macular ganglion cell layer volume, mm^3^
Disease duration	−	+/−	+	+ +	−
Diagnostic LP opening pressure	−	−	−	+ +	−
Baseline BMI	−	−	−	−	+
Change in BMI	−	+	+ + +	+/−	+ + + +
ICP medication During v never	−	−	−	−	−

(− *p* > 0.08, +/− *p* = 0.05–0.08, + *p* ≤ 0.05, +  + *p* ≤ 0.01, +  +  + *p* ≤ 0.001, +  +  +  + *p* ≤ 0.0001)

### Factors affecting long-term headache outcomes

**Fig. 4:**
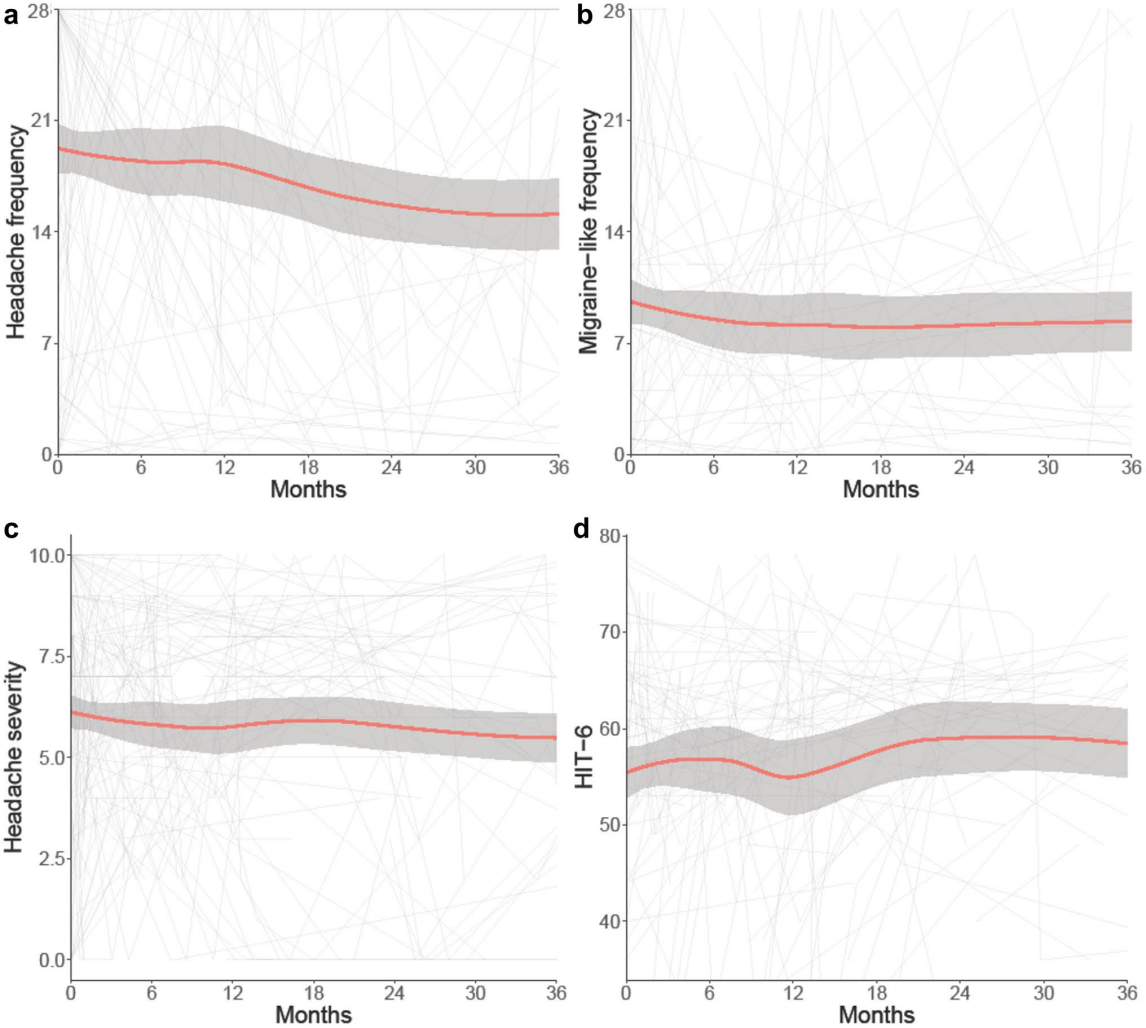
Longitudinal headache data from medically managed IIH patients with active disease (papilloedema present at enrolment), and LOESS smoothers added to show trends across the categories. **a** Headache frequency (days per month). **b** Migraine-like headache frequency (days per month). **c** Headache mean severity of predominant headache (0–10 numerical rating scale). **d** Headache Impact Test 6 (HIT6) (quality of life measure score 36–78)

In the medically managed patients (*n* = 281) there was a high burden of headache (Table 3). The mean headache frequency at baseline was 21 days/month (SD 12.2), which improved over time, with stable migraine-like headache frequency, headache severity and headache disability (Table 3). As a result of sparse headache data in the ocular remission cohort, we focussed our inferences on the medically managed cohort with papilloedema (active disease) at the first encounter (Fig. 4).

Headache frequency showed a more rapid rate of improvement in this active group as compared to the whole cohort (− 0.46 days per month/month (95% CI − 0.67, − 0.25), versus − 0.20 days per month/month (95% CI − 0.28, − 0.12)). Stepwise regression analysis showed that the only factors affecting long-term headache frequency were the occurrence of daily headache at diagnosis and a personal migraine history. Disease duration, change in BMI and family history of migraine were not significantly influential (Table 6).

**Table 6:** Factors affecting headache prognosis in medically managed active IIH cohort

	Headache frequency	Migraine-like headache frequency	Headache severity	Headache Impact Test 6
Disease duration	−	−	+ +	−
Change in BMI	+/−	−	−	−
Daily headache at baseline	+ + + +	+ + + +	+ + + +	−
Migraine Personal history	+ + + +	+ + + +	+ + + +	−
Migraine Family history	−	−	+	−
Analgesic overuse	−	−	−	−
ICP medication During v never	−	−	−	−

(− *p* > 0.08, +/− *p* = 0.05–0.08, + *p* ≤ 0.05, +  + *p* ≤ 0.01, +  +  + *p* ≤ 0.001, +  +  +  + *p* ≤ 0.0001)

Monthly migraine-like headache days occurred with a frequency of 8.14 days/month (95% CI 7.09, 9.18) at baseline. There was a relatively little improvement over time [− 0.06 days per month/month (95% CI − 0.12, − 0.01)] (Fig. 4). The only factors affecting migraine-like headache frequency outcomes were personal migraine history (higher baseline frequency by 4.7 days/month (95% CI 2.38, 6.98) and trajectory − 0.1 days per month/month (95% CI − 0.28, 0.02)) and daily headache at diagnosis [higher baseline frequency by 4.2 days/month (95% CI 2.32, 6.13)]. There was no significant effect of disease duration, BMI, familial history of migraine or medication overuse (Table 6).

Headache severity was reported as moderate at the first visit [6.1 out of 10 (95% CI 5.74, 6.40)] and remained stable (Fig. 4). A personal migraine history was a poor prognostic indicator as it increased headache severity (increase of 1.6 units (95% CI 0.76, 2.35)). To a lesser extent disease duration affected the prognosis by increasing headache severity by 0.03 per year of disease duration (95% CI 0.008, 0.043) (Table 6).

Overall headache disability, as measured by the HIT-6, was high at the first visit with a mean score 56.47 (95% CI 54.58, 58.37) and showed no significant improvement over time (0.08 units per month (95% CI − 0.04, 0.21), Fig. 4). None of the factors explored affected longitudinal HIT-6 scores (Table 6).

## Discussion

In this study, those with the greatest papilloedema, RNFL > 400 µm or TRT ≥ 800 µm, had the greatest loss of macular GCL volume. Importantly there was a delay of over 12 months from the baseline visit before the visual field and OCT measurements revealed this decline. Increases in BMI and disease duration had the most influence on visual prognosis. This potentially suggests that preventing high grades of papilloedema and modifying BMI early may be beneficial for long-term visual outcomes. Headache outcomes showed marked heterogeneity in this cohort, with regression modelling showing a personal migraine history and daily headache at diagnosis were the principal factors influencing the worst headache prognosis. For those that presented in ocular remission, the disease course was favourable with only two cases having relapse, both of which were associated with weight gain.

In those with active papilloedema at enrolment, OCT RNFL improved over time (Fig. 3 and Table 4) and the literature would support these findings [10]. Automated RNFL thickness measures may be compromised in moderate to severe papilloedema and accurate segmentation is essential. We recognise that this can be time consuming in busy clinical practice when the automated segmentation fails [34, 39–42]. As part of this study we chose to manually segment all the individual OCTs to denote RNFL and we elected to manually produce a TRT measurement derived from the RNFL scan, where required. Our results for TRT are similar to those for RNFL, which is important evidence for the integration of TRT in preference to RNFL into clinical practice, due to greater accuracy in moderate to severe papilloedema. OCT may be a more sensitive marker for disease status than measures of visual function, such as PMD, and is a more objective measure than Frisén grading [32, 33]. A positive association between intracranial pressure and OCT central thickness and RNFL has been previously demonstrated, highlighting the use of OCT as surrogate marker for intracranial pressure measurements [33]. In those patients who presented in ocular remission and demonstrated substantially thinner RNFL at follow-up, it is likely that their intracranial pressure had settled, as compared to those who presented with active disease [13].

Macular GCL thickness is a measure of neuronal axonal loss[43] and is positively correlated with visual field loss at 12 months[33] and following papilloedema resolution [44]. Progressive decline over 12 months in both RNFL and GCL has previously been shown in acute optic neuritis [45] highlighting that OCT measures change over a longer time frame, reflecting the long-term term fall out of optic neuropathy. Our findings here in IIH, showed a decline in GCL volume in those with severe papilloedema (as defined by an RNFL ≥ 400 µm or TRT ≥ 800 µm, Fig. 2) with a corollary decline in PMD also noted. This decline in parameters was noted after 12 months, thus indicating the need for long term follow up and monitoring of patients who previously had severe papilloedema. This difference may be because severe IIH has exerted a greater intracranial pressure on the optic nerve, leading to ischaemia, causing a worse optic neuropathy and subsequent atrophy [46]; often by the time surgical intervention occurs the damage may be evidently irreversible. Future work needs to define what clinical biomarkers could be used to ensure timely intervention and reduction of this long-term damage [47].

For the majority, visual field PMD showed mild improvement, interestingly there was little difference in trajectory between the active and remission groups (those with and without papilloedema at enrolment). Our cohort’s improvement is more modest than previously presented longitudinal data but has the advantage of being in a prospective and much larger cohort [10]. Our PMD plateau was very similar to that seen in the IIH Treatment Trial, at approximately − 2 dB [20]. Beyond 18 months the mean PMD was stable in the majority of our cohort, which is reassuring for patients, but also indicates that if there is a clinically relevant deterioration during follow-up it may be important to screen for relapse or investigate for another non-related pathology.

IIH is well known to be associated with obesity [3]. Obesity is likely to be one manifestation of the underlying metabolic disease with adipocytes being transcriptionally and metabolically primed towards lipogenesis and adipose accumulation [1]. Weight reduction is challenging but appears to be the only treatment so far found to modify disease course in IIH [15, 16]. Here we found change in BMI was the most important factor to influence visual outcomes. Increasing BMI during follow-up was shown to be associated with the worsening of OCT RNFL and PMD, highlighting the importance of weight management in IIH patients and is an ongoing area of research [3]. Our relapse rate was 3.7% in the whole cohort and 1.4% in those in ocular remission at enrolment, which is lower than previously reported in the literature, and this may reflect the increasing evidence and awareness of the importance of weight management in this disease [3, 15, 16].

Headache is a common symptom in the IIH population, which was also reflected in this cohort, with a high baseline HIT-6 score [12, 14]. The trajectory for headache frequency showed a slow improvement over time, likely reflecting identification, and treatment. In this analysis, the two main factors influencing high headache frequency and worse prognosis were a personal migraine history and daily headache at baseline. This has previously not been demonstrated. Therefore, it may be important to adjust for migraine history and daily headache in future IIH headache trials as they may influence clinical trial outcomes [48]. Interestingly regression analysis did not show that medication overuse alone influenced headache frequency in IIH, however, in the real world there are multiple interconnected factors that can contribute to headache, one of which may be medication overuse and we only assessed medication overuse particularly in chronic headache disorders. There are however disputes as to whether medication overuse is a cause or consequence of the chronification of headaches [49]. The advent of CGRP therapy may impact headache outcomes in IIH in the future [50–52].

This was a prospectively collected study of real-world clinical practice and has some inherent limitations. As it is within a tertiary referral centre, a number of patients from the cohort would have been seen initially at other hospitals. Whilst neuroimaging is requested for review, the clinical details are not routinely requested, other than what is stated in the referral letter. Therefore, baseline data for each participant could have been at a different point in their actual disease course. To reduce this bias, we defined disease duration from the LP to the first encounter in our clinic. We also recognise that patients report symptoms of IIH in a variable time frame before an actual diagnosis. Although the study aimed to capture all outcomes, some were missing due to patient preference. Also, as per clinical practice some individuals who presented in ocular remission were discharged, some were lost to follow-up, and hence these groups had no further clinic appointments or data collected. This resulted in a reduction in the number of patients over the course of the study. We, therefore, advise caution in over interpreting the long-term outcomes as seen in the LOESS smoothers curve due to the reducing sample size over the course of the study. To reduce the bias of missing data, we elected to only analyse headache outcomes in those with active papilloedema, rather than those who presented in remission, as this latter group were more likely to be discharged and had less follow-up data. Likewise, due to the challenges of data collection for intracranial pressure lowering medications, such as confirming dose, duration, and inability to ensure compliance, we only reported baseline data and did not further analyse their effects on outcomes here. This was also the case for analgesic frequency. In the future, our IIH Life study may collect more detailed descriptions of medications and other factors. In addition, thefuture analysis could be compared to a control group which may allow for more insights into the long-term outcomes of IIH to be discovered.

## Conclusions

In this prospective real-world longitudinal cohort of individuals with IIH presenting to a tertiary referral centre, the outcomes were generally good. However, decline in PMD and macular GCL volume is delayed and seen during follow-up beyond 12 months. A minority of patients required sight-saving surgery, and generally had worse long-term outcomes for their visual parameters. The headache burden was high in this cohort and prognosis was impacted by previous migraine diagnosis and occurrence of daily headaches at the first visit. Targeted headache treatment remains an unmet clinical need. Body weight modification and disease duration appear to have the main influence for visual outcomes, and hence why we would recommend active early weight management for patients with IIH with a raised BMI.

## Data Availability

Professor Sinclair takes full responsibility for the data, the analyses and interpretation, and the conduct of the research. She has full access to all the data; and has the right to publish any and all data separate and apart from any sponsor. Proposals for data access should be made to the corresponding author. Reasonable scientifically sound proposals, from appropriately qualified research groups, will provide data beginning 12 months and ending 3 years after the publication of this article to researchers whose proposed use of the data is approved by the corresponding author. Requesters will need to sign a data access agreement, which will cover the terms and conditions of the release of data and will include publication requirements, authorship, acknowledgements, and obligations for the responsible use of data.

## Declarations

### Disclaimer

The study sponsor and funders had no role in the study design, interpretation of the data, data collection, or drafting of the manuscript.
